# Combined Cytotoxic Effects of the Fungicide Azoxystrobin and Common Food-Contaminating Mycotoxins

**DOI:** 10.3390/foods14071226

**Published:** 2025-03-31

**Authors:** Cristina Fuentes, Veronica Zingales, José Manuel Barat, María-José Ruiz

**Affiliations:** 1 University Institute of Food Engineering–FoodUPV, Universitat Politècnica de València, Camino de Vera s/n, 46022 Valencia, Spain; jmbarat@tal.upv.es; 2 Research Group in Alternative Methods for Determining Toxics Effects and Risk Assessment of Contaminants and Mixtures (RiskTox), Universitat de València, Av. Vicent Andrés Estellés s/n, 46100 Burjassot, Spain; veronica.zingales@uv.es (V.Z.); m.jose.ruiz@uv.es (M.-J.R.); 3 Laboratory of Food Chemistry and Toxicology, Faculty of Pharmacy, Universitat de València, Av. Vicent Andrés Estellés s/n, 46100 Burjassot, Spain

**Keywords:** fungicide, mycotoxins, chemical mixtures, interaction, HepG2 cells

## Abstract

This study assessed the cytotoxicity of the individual and combined exposure to the fungicide azoxystrobin (AZX) and the three common mycotoxins found in food: ochratoxin A (OTA), deoxynivalenol (DON), and T-2 toxin. Cytotoxic effects were evaluated using the resazurin and MTT assays in human hepatocarcinoma (HepG2) cells after 24 h of exposure, and the type of interaction between the compounds was determined using the isobologram method. Results showed that T-2 was the most cytotoxic compound, followed by DON, OTA, and AZX. The compound ratios in the mixture were calculated using three sublethal concentrations (IC_50/2_, IC_50/4_, and IC_50/8_) to achieve equal toxicity for each compound. Interaction analysis revealed that the nature of the interaction varied across components and concentrations. The AZX and DON mixture produced an antagonistic effect at all the analyzed effect levels. AZX and OTA or T2 mixtures, and tertiary combinations displayed antagonism at low effect values but additivity at high effect levels. Importantly, the quaternary mixture demonstrated synergism at all the effect levels. These findings highlight that the co-occurrence of fungicides and mycotoxins in food commodities can lead to complex exposure scenarios that may result in combined toxic effects on the organism.

## 1. Introduction

In agriculture, fungicides are essential for safeguarding crops from fungal diseases that cause significant yield reductions worldwide. They are applied directly to ornamental plants, trees, field crops, cereals, and turf grasses, and are used to protect tubers, seeds, fruits, and vegetables during storage [1]. Fungicides represent one of the most extensively utilized categories of pesticides and play a critical role in safeguarding the global food supply and ensuring food safety. In 2022, “fungicides and bactericides” comprised the largest pesticide sales in the EU, at a percentage of 43% [2]. Moreover, the global fungicides market is expected to experience a 4.93% Compound Annual Growth Rate (CAGR) from 2024 to 2032 [3]. This increase is expected due to the rising need for higher agricultural yields to support a growing global population, increasing global temperatures and shifting weather patterns, which result in higher rates of fungal diseases in crops [4].

Although fungicides can prevent crop diseases and increase yields, their widespread use may expose consumers to these compounds through various diet-related commodities. To ensure a high level of consumer protection, Regulation (EC) No 396/2005 of the European Parliament and the Council of 23 February 2005 established maximum residue levels (MRLs) in food or animal feed of plant and animal origin based on the most critical good agricultural practice (GAP) and the lowest exposure necessary to protect vulnerable consumers [5]. According to the latest EFSA annual report on pesticide residues in food, out of a total of 110,829 food samples analyzed from the European market, 96.3% were below the MRLs, and only 3.7% exceeded this level, with 2.2% being non-compliant [6]. The MRLs established in the EU legislation are based on the potentially toxic effects of individual exposure to these chemicals and do not consider the presence of different classes of chemicals simultaneously [7]. Notwithstanding, combined exposure to multiple xenobiotics through diet raises concerns because substances can interact synergistically or additively, enhancing their toxic effects, which may exceed the individual risks of each compound [8]. Such interactions can lead to unpredictable health outcomes, including increased toxicity, reproductive and developmental disturbances, immune system impairment, or even cancer and neurodegenerative diseases [9,10], making it challenging to assess safe exposure levels and try to mitigate potential long-term health risks. In 2012, the European Commission published a communication on the combined effects of chemicals, expressing concern about the limitations of evaluating compounds individually and the need to study combined exposure to multiple chemicals [11]. Since then, institutions and researchers have been developing new approaches and tools to harmonize the study of chemical mixtures. The goal is to gather toxicological information from simultaneous exposure to multiple substances that may be found in the food chain. However, very little information is available to date since different gaps make their study difficult, including a limited understanding of how chemicals interact in mixtures, the infinite number of chemical combinations, the complexity of evaluating the effects of long-term or chronic low-level exposure, or the lack of standardized testing and biomonitoring methods for assessing combined exposures [12].

Azoxystrobin (AZX) is one of the most used active ingredients in fungicide products worldwide [13]. It is a systemic fungicide with broad-spectrum activity, effective against all four major taxonomic groups of fungi responsible for fungal diseases: *Ascomycota*, *Deuteromycota*, *Basidiomycota*, and *Oomycota* [14]. AZX is approved for use on a wide range of crops in the EU, including fruits, vegetables, small grains, and turf grass. In particular, it is commonly used on wine grapes, coffee, cotton, sugarcane, potatoes, apples, bananas, broccoli, cauliflower, rice, almonds, pistachios, barley, soybean, or wheat, among other crops [15,16,17].

One of the major risks of fungal contamination of vegetable products is the presence of mycotoxins. These are chemical compounds produced by the secondary metabolism of some genera of fungi, such as *Aspergillus*, *Penicillium*, *Fusarium*, *Claviceps*, and *Alternaria*, which contaminate foods such as cereals, fruits and nuts, oilseeds, spices, and feed [18]. Various mycotoxins can affect human and animal health, with deoxynivalenol (DON), ochratoxin A (OTA), and T-2 toxin (T2) being among the most identified in food and feed. Long-term exposure to these and other mycotoxins can lead to adverse health effects, including kidney and gastrointestinal problems, alterations in the immune system or estrogen metabolism, cancer development, and mutagenesis [19].

Laboratory studies and field data have highlighted the variable efficacy of fungicides in controlling mycotoxin residues in cereals and grains harvested from crops due to the acquisition of fungicide resistance [20]. Resistance to fungicides can develop due to genetic mutations, modifications at target sites, overexpression of target enzymes, activation of efflux pumps, and metabolic detoxification [21]. These adaptations enable pathogens to survive chemical treatments and continue to proliferate. The rise of resistance in essential classes of fungicides poses significant challenges to control efforts, often leading to increased chemical application [22]. This situation raises potential health risks for humans and animals due to exposure to contaminated food and water sources. In this respect, various studies have demonstrated the co-occurrence of AZX and mycotoxins in several food products. Giorni et al. (2019) studied the efficacy of AZX in reducing the presence of mycotoxigenic fungi and relative mycotoxins in Italian paddy rice during the growing season in the field. These authors found that treatment with AZX was ineffective in reducing DON contamination, as 46% of samples contained DON at levels lower than 100 µg/kg [23]. Palladino et al. (2021) determined the concentration level of *Fusarium* mycotoxins and fungicide residues in 89 barley grain samples from different commercial pads. The analysis revealed that 74% of the grain samples contained fungicide residues, all below their corresponding MRLs. According to this study, AZX was the most frequently found fungicide, and DON was the most detected mycotoxin, present in 88 out of the 89 samples, and of these, 31% exceeded the MRLs [24].

The co-occurrence of AZX and mycotoxins in food products can lead to complex exposure scenarios, resulting in combined toxic effects that may cause detrimental effects on the organism [25]. This highlights the growing need to understand the combined toxicity of these chemicals for a more accurate assessment of human health risks [26]. Consequently, this study aimed to assess the in vitro toxicity from the combined exposure to the fungicide AZX and three common mycotoxins found in food. Specifically, the cytotoxic effect of exposure to AZX alone or combined with ochratoxin A (OTA), DON, and T-2 toxin (T2) was investigated in the human hepatocarcinoma (HepG2) cell line. In addition, the interaction between these compounds in different combinations has been determined.

## 2. Materials and Methods

### 2.1. Reagents

The human hepatocarcinoma cell line HepG2 was purchased from the American Type Culture Collection (ATCC HB-8065). Dulbecco’s Modified Eagle Medium (DMEM-GlutamaxTM) with high glucose (4.5 g/L), Phosphate-Buffered Saline (PBS), Newborn Calf Serum (NBCS), penicillin, streptomycin, and trypsin-EDTA 0.5% were obtained from Gibco (Thermo Fisher Scientific, Waltham, MA, USA). Dimethyl sulfoxide (DMSO) and methanol were acquired from Fisher Scientific (Madrid, Spain). The standards of AZX (purity ≥ 98%, MW 403.39 g/mol), DON (purity ≥ 98%, 296.32 MW g/mol), OTA (purity ≥ 97%, MW 403.81 g/mol), T2 (purity ≥ 98%, MW 466.52 g/mol), thiazolyl blue tetrazolium bromide (MTT), and the resazurin sodium salt were supplied by Sigma-Aldrich (St Louis, MO, USA). All the reagents and cell culture components were of standard laboratory grade.

The fungicide and the mycotoxins were prepared as stock solutions in DMSO and methanol, respectively. These solutions were stored at −20 °C until use. Working concentrations were then prepared in a DMEM-supplement medium with a maximum solvent concentration of 0.1% in the test solutions. Negative controls containing the appropriate amounts of solvents were included in every experiment.

### 2.2. Cell Culture

The HepG2 cells were cultured in DMEM-Glutamax medium completed with 10% NBCS, 100 U/mL penicillin, and 100 µg/mL streptomycin. Cells were grown in monolayer culture flasks at pH 7.4, 5% CO_2_ at 37 °C, and 95% air atmosphere at constant humidity. The culture medium was changed every 2 days. Cell subculturing was performed twice weekly (<30 subcultures to guarantee genetic homogeneity) by treating the cells with trypsin-EDTA for 3 min at 37 °C in a 1:3 split ratio. The absence of mycoplasma was periodically examined using the MycoAlertTM PLUS Mycoplasma Kit (Lonza, Rockland, ME, USA).

### 2.3. In Vitro Cytotoxicity

Cytotoxic effects were evaluated in HepG2 cells using the MTT and the resazurin reduction assays. Both assays were performed simultaneously on the same plate as Efeoglu et al. (2017) described [27]. Briefly, 96-well culture plates were seeded by adding 100 µL per well of a cell suspension of 1 × 10^5^ cells/mL and incubated at 37 °C in a 5% CO_2_ atmosphere for 24 h. After this time, wells were washed with 100 µL PBS, and the cells were treated with 100 µL of serial dilutions of the test solutions. Different concentrations of AZX (78–1000 µM), DON (2.81–30 µM), OTA (1.91–245 µM), and T-2 (9.38–100 nM) were tested. Following the exposure period, wells were washed with 100 µL PBS, and 100 µL of non-supplemented DMEM medium with 10% MTT (5 mg/mL in PBS) and 5% resazurin (0.25 mg/mL) were added. Then, plates were wrapped in foil and incubated at 37 °C in a 5% CO_2_ atmosphere for 3 h. After incubation, a multimode microplate reader quantified the resorufin fluorescence (λexcitation = 560 nm, λemission = 590 nm) (Biotek Synergy H1, BioTek Instruments, Winooski, VT, USA). Next, wells were washed with PBS and filled with 100 µL DMSO to solubilize the formazan crystals. Finally, plates were shaken at 240 rpm for 5 min, and the absorbance was measured at a wavelength of 570 nm in a Multiskan EX ELISA plate reader (Thermo Fisher Scientific, Waltham, MA, USA). Cell viability was expressed as a percentage of non-treated cells (negative control).

### 2.4. Experimental Design and Analysis of the Interaction of Chemical Mixtures

The cytotoxic effects of the mixture of AZX with the mycotoxins DON, OTA, and T2 were determined using the resazurin and the MMT methods. The effects were tested in three binary combinations (AZX + DON, AZX + OTA, and AZX + T2), three tertiary combinations (AZX + DON + OTA, AZX + DON + T2, and AZX + OTA + T2), and a quaternary combination (AZX + DON + OTA + T2). All the cytotoxic assays were performed after 24 h of exposure. The combination ratios of each compound in the mixture were calculated based on the IC_50_ values obtained in the MTT individual cytotoxicity experiments. These combination ratios were chosen to obtain equipotent toxicity for each compound. In particular, three sublethal concentrations (IC_50/2_, IC_50/4_, and IC_50/8_) combined at a fixed constant ratio were examined. The concentrations of each compound and the combination ratios used in this study of the chemical mixtures are shown in Table 1.

The data obtained in the cytotoxicity tests were used to analyze the nature of the interactions between AZX and the mycotoxins DON, OTA, and T2 using the median–effect/combination index (CI)–isobologram model developed by Chou and Talalay [28]. According to this model, dose–effect curves for each compound and their combinations at different concentrations were generated using the median–effect equation as follows:

(1) $$\frac{fa}{fu}={\left(\frac{D}{Dm}\right)}^{m},$$

where *D* represents the concentration, *fa* is the fraction affected by *D*, *fu* is the unaffected fraction (*fu* = 1 − *fa*), *Dm* is the median–effect concentration (concentration required to cause a 50% effect), and m represents the Hill coefficient that characterizes the shape of the dose–effect curve: *m* = 1, *m* > 1, and *m* < 1 correspond to hyperbolic, sigmoidal, and negative sigmoidal curves, respectively [28].

The assessment of compound interactions within the mixture was conducted using combination index (CI) values at an inhibition rate of 10%, 25%, 50%, 75%, and 90%, generated as follows:

(2) $${\left(CI\right)}_{x}=\sum_{j=1}^{n}\left(\frac{{\left(D\right)}_{j}}{{\left(D_{x}\right)}_{j}}\right)=\sum_{j=1}^{n}\frac{{\left(D_{x}\right)}_{1-n}\left\{\frac{{\left[D\right]}_{j}}{\sum_{1}^{n}\left[D\right]}\right\}}{{\left(D_{x}\right)}_{j}{\left\{\frac{{\left(fax\right)}_{j}}{\left[1-{\left(fax\right)}_{j}\right]}\right\}}^{1/mj}},$$

where (*CI*)*_x_* represents the combination index for compounds at *x*% inhibition effect, (*D_x_*)_1−*n*_ is the sum of the concentrations of *n* compounds that cause *x*% inhibition effect in the mixture, {[*D*]*_j_/*$\sum_{1}^{n}\left[D\right]$ is the proportionality of a specific concentration of each compound that causes *x*% inhibition level in combination, and (*D_x_*)*_j_*{(*fax*)*_j_*/1 − (*fax*)*_j_*}^1/*mj*^ refers to the concentration of each compound that produces *x*% inhibition. Based on the combination index, this equation categorizes compound interactions as synergistic (CI < 1), antagonistic (CI > 1), or additive (CI = 1). The isobologram analysis was performed using CalcuSyn software version 2.1 (Biosoft, Cambridge, UK, 1996–2007).

### 2.5. Statistical Analysis

The statistical analysis was performed using the Statgraphics Centurion XVI software package, version 16.2.04 (Statgraphics Technologies, Inc., The Plains, VA, USA). Data were expressed as the mean of at least three independent experiments (mean ± SEM). Each compound’s mean inhibition concentration (IC_50_) values were estimated using a four-parameter sigmoidal fit in GraphPad Prism software, version 8.0.1 (GraphPad Software, Boston, MA, USA). A Student’s *t*-test statically analyzed the results for paired samples.

## 3. Results

### 3.1. Cytotoxicity of Individual Components

**Figure 1:**
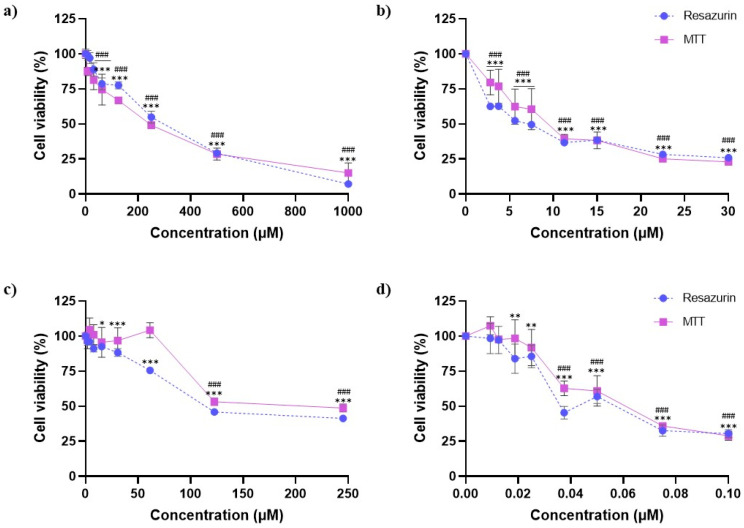
Cell viability–concentration curves of HepG2 cells exposed to (**a**) AZX, (**b**) DON, (**c**) OTA, and (**d**) T2 for 24 h as measured using the resazurin (blue) and the MTT (purple) assays. Bars represent the mean (SEM) of three independent assays, each performed six-fold. Student’s *t*-test was used to analyze statistically significant differences between the treatments and the control. (*) *p* ≤ 0.05; (**) *p* ≤ 0.01; and (***) *p* ≤ 0.001 indicate significant differences in the resazurin assay. (###) *p* ≤ 0.001 indicate significant differences in the MTT assay.

The cytotoxic effect of individual exposure to AZX and the mycotoxins DON, OTA, and T2 was evaluated at 24 h exposure using the resazurin and MTT assays. Figure 1 shows the cell viability–concentration curves for the different substances. All the compounds reduced cell viability in a concentration-dependent manner. As observed, T2 was the most cytotoxic compound, followed by DON, OTA, and AZX in that order. HepG2 cell viability decreased from 30% to 98% after 24 h of exposure to T2 concentrations ranging from 0.013 µM to 0.100 µM using both methods. DON exposure diminished cell viability from 26% to 63% using the MTT assay and from 23% to 80% using the resazurin method at the 2.8–30 µM concentration range. A reduction in cell viability from 41% to 96% was observed after 24 h exposure to OTA (1.9–245 µM) using the MTT method and from 49% to 97% using the resazurin assay. Similarly, AZX significantly reduced HepG2 cell viability, with decreases ranging from 7% to 99% and 15% to 87% at concentrations from 7.8 µM to 1000 µM, as measured using the MTT and resazurin assays.

Small differences were found in the IC_50_ values obtained from the resazurin and MTT assays. The IC_50_ values for T-2 were 0.051 (0.012) µM and 0.059 (0.013) µM, as determined from the resazurin and MTT assays, respectively. The IC_50_ values for DON were 6.61 (0.56) µM, as determined by the resazurin method, and 9.39 (1.96) µM, as determined by the MTT assay. The IC_50_ values for OTA after HepG2 cell exposure were 147.3 (28.5) µM and 195.3 (76.7) µM, as determined using the resazurin and MTT methods, respectively. Finally, AZX, the least cytotoxic of the compounds analyzed, showed IC_50_ values of 231.2 (41.2) µM and 206.1 (49.9) µM according to the resazurin and MTT assays, respectively.

### 3.2. Cytotoxicity of AZX and Mycotoxins Combinations

**Figure 2:**
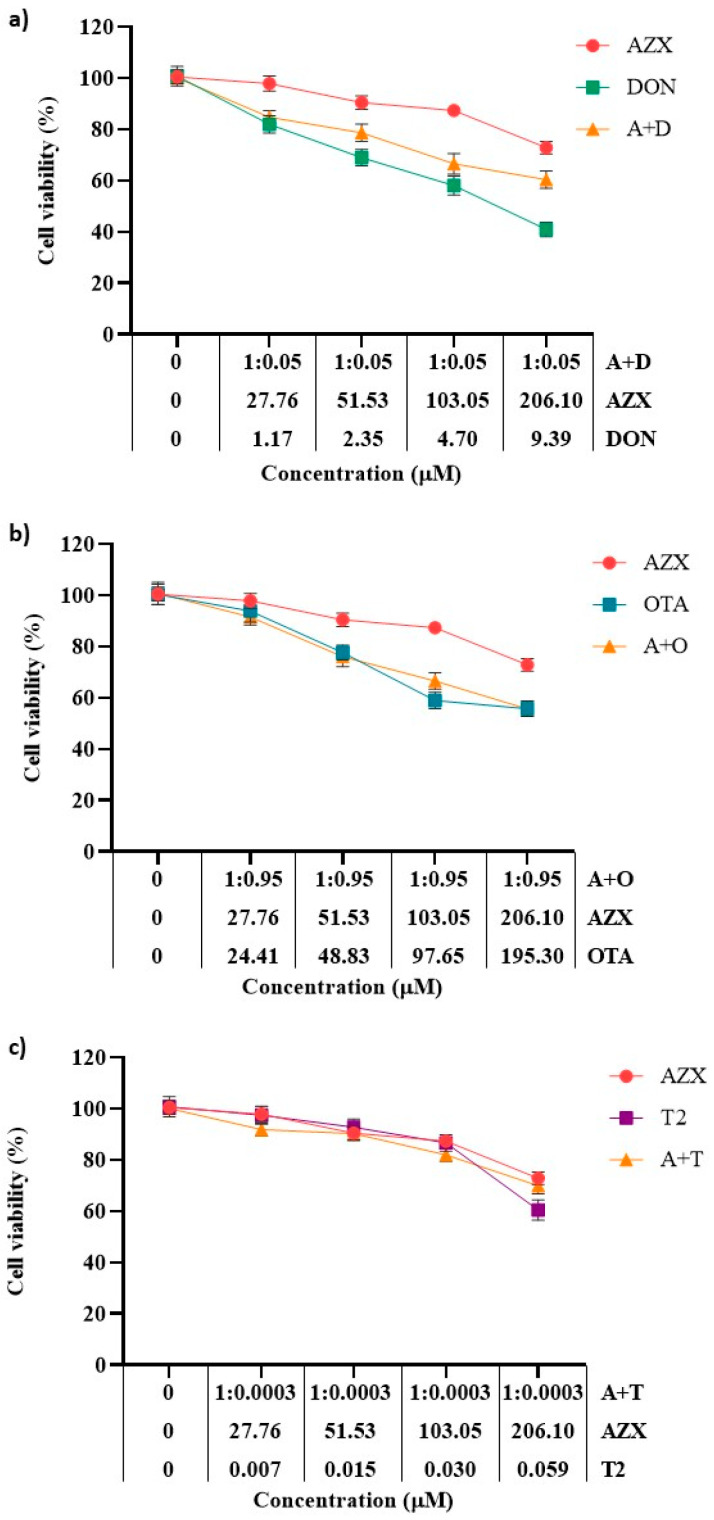
Cell viability–concentration curves of HepG2 cells exposed to individual and binary combinations of (**a**) AZX and DON, (**b**) AZX and OTA, and (**c**) AZX and T2 for 24 h in HepG2 cells, as measured using the resazurin assay. A and AZX: azoxystrobin; T and T2: toxin T2; O and OTA: ochratoxin A; and D and DON: deoxynivalenol.

**Figure 3:**
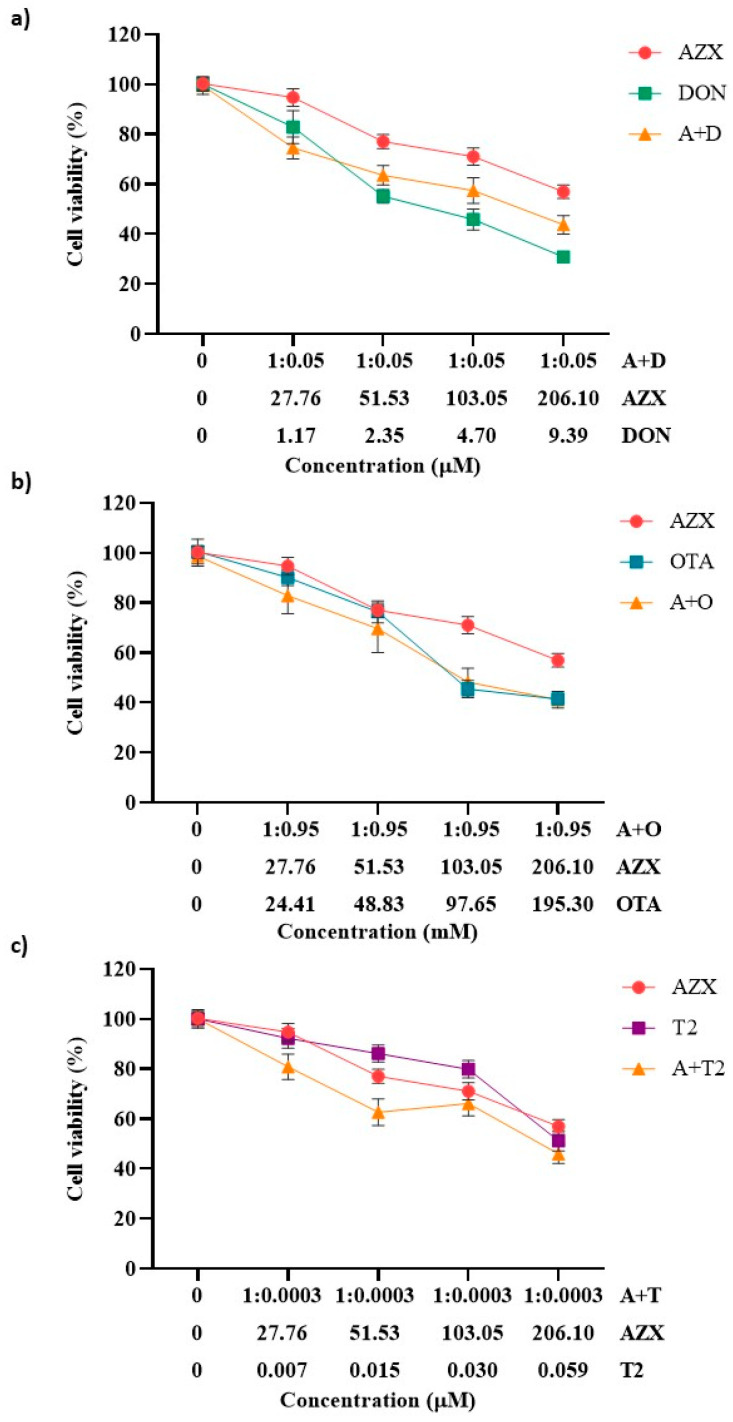
Cell viability–concentration curves of HepG2 cells exposed to individual and binary combinations of (**a**) AZX and DON, (**b**) AZX and OTA, and (**c**) AZX and T2 for 24 h in HepG2 cells as measured using the MTT assay. A and AZX: azoxystrobin; T and T2: toxin T2; O and OTA: ochratoxin A; D and DON: deoxynivalenol.

Figure 2 and Figure 3 show the effects of the binary combinations of AZX with the mycotoxins DON, OTA, or T2 (AZX + DON, AZX + OTA, and AZX + T2) on cell viability, as determined by the resazurin and MTT assays, respectively. Significant differences were found between the mixture and the individual compounds. The mixture AZX + DON (Figure 2a and Figure 3a) resulted in a higher cell viability reduction than AZX when administered alone, but lower than that of DON individually. At the higher concentration tested, AZX + DON reduced cell viability by 40%, DON by 59%, and AZX by 27% according to the resazurin method, and by 56%, 69%, and 43%, respectively, according to the MTT assay. The mixture AZX + OTA (Figure 2b and Figure 3b) reduced cell viability similarly to OTA but to a greater extent than AZX alone. AZX + OTA and OTA showed a reduction percentage of 44% and 59% at higher concentrations under the resazurin and MTT assays, respectively, and 27% and 43% in the case of AZX. The mixture AZX + T2 (Figure 2c and Figure 3c) showed a similar effect on HepG2 viability at the maximum concentration tested compared to AZX (30% and 27%, respectively), but lower than T2 (40%) under the resazurin method. Instead, when using the MTT method, no significant differences were observed at the higher concentration tested between the binary mixture AZX + T2 and AZX and T2 individually (with reduction percentages ranging between 43% and 54%). However, the mixture AZX + T2 resulted in greater cytotoxic effects than AZX and T2 alone, as determined by this method at EC50/2 and EC50/4 concentrations (Figure 3c).

**Figure 4:**
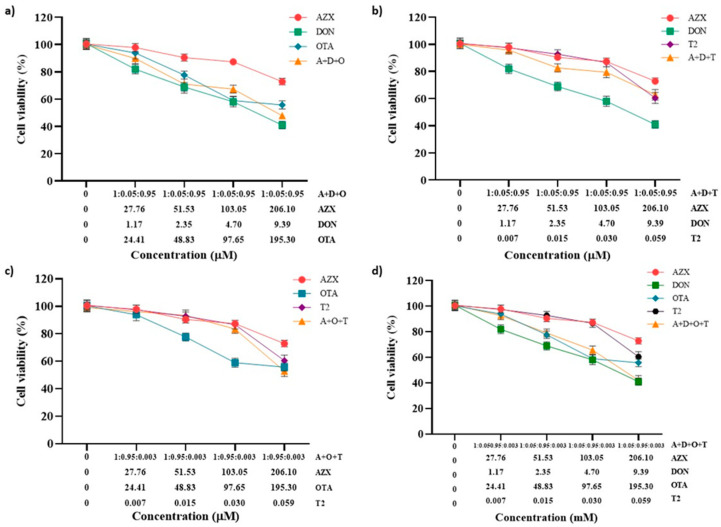
Cell viability–concentration curves of HepG2 cells exposed to individual and tertiary combinations of (**a**) AZX, DON, and OTA, (**b**) AZX, DON, and T2, (**c**) AZX, OTA, and T2, and (**d**) the quaternary combination of AZX, DON, OTA, and T2 for 24 h in HepG2 cells, as measured by the resazurin assay. A and AZX: azoxystrobin; T and T2: toxin T2; O and OTA: ochratoxin A; D and DON: deoxynivalenol.

**Figure 5:**
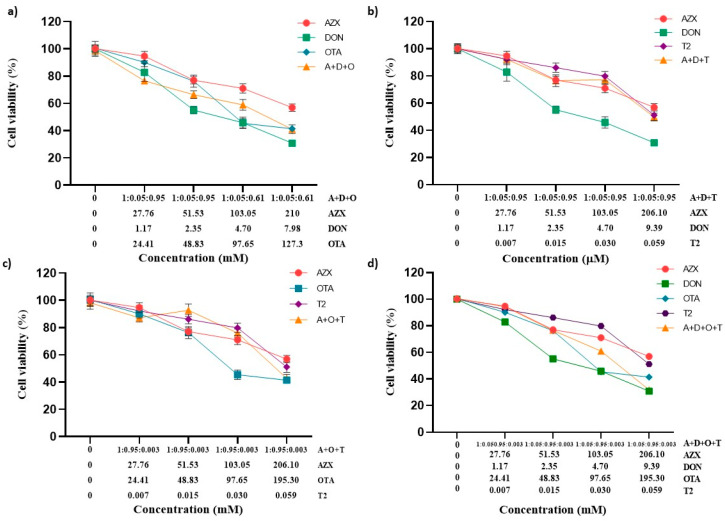
Cell viability–concentration curves of HepG2 cells exposed to individual and tertiary combinations of (**a**) AZX, DON, and OTA, (**b**) AZX, DON, and T2, (**c**) AZX, OTA, and T2 for 24 h in HepG2 cells as measured using the MTT assay, and (**d**) the quaternary combination of AZX, DON, OTA, and T2 for 24 h in HepG2 cells as measured by the resazurin assay. A and AZX: azoxystrobin; T and T2: toxin T2; O and OTA: ochratoxin A; D and DON: deoxynivalenol.

The effect of the tertiary combinations of AZX and the mycotoxins DON, OTA, and T2 on HepG2 viability, as assessed using the resazurin and MTT methods, is shown in Figure 4a–c and Figure 5a–c, respectively. The tertiary mixture AZX + DON + OTA resulted in higher cytotoxic effects than AZX when administered alone. As measured by the resazurin assay, the tertiary combination AZX + DON + OTA decreased cell viability by 8% to 25% more than AZX alone (Figure 4a). According to the MTT method, the reduction range was 11% to 18% higher for the combination (Figure 5a). In contrast, smaller differences were observed by both methods between the tertiary mixture and DON or OTA individually at the various concentrations tested. The mixture AZX + DON + T2 showed small or no differences compared to AZX and DON individually, depending on the concentration tested, while DON resulted in higher reduction percentages than the combination treatment. DON decreased cell viability by 14% to 22% more than AZX + DON + T2, as measured by the resazurin method, and by 9% to 21% according to the MTT assay. The AZX + OTA + T2 combination resulted in a lower cytotoxic effect than OTA alone at EC50/2, EC50/4, and EC50/8 concentrations, with reductions ranging from 4% to 13%, as determined by the resazurin method, and from 13% to 27%, as determined by the MTT method. At the highest concentrations tested, AZX + OTA + T2 only displayed differences when compared to AZX individually, with the mixture showing a reduction percentage that was 20% higher.

Figure 4d represents the cytotoxic effect of exposure to the quaternary combination AZX + DON + OTA + T2, as measured using the resazurin method. The quaternary mixture was more cytotoxic than AZX and T2 at the three higher concentrations (51.53–206.10 µM for AZX and 0.015–0.059 µM for T2). By contrast, the four-component combination was more cytotoxic than DON at the same concentrations (2.35–9.39 µM), and more cytotoxic than OTA at the highest concentration assayed (195.3 µM). Similar results were found with the MTT assay (Figure 5d). The quaternary mixture at the three highest concentrations analyzed resulted in higher cytotoxic effects than AZX and T2 but lower than OTA. DON produced higher cytotoxicity than the mixture at the three lowest concentrations (2.35–9.39 µM).

### 3.3. Toxicological Interactions Between AZX and Mycotoxins

The isobologram analysis was performed to determine the type of interaction between three binary combinations, three tertiary combinations, and a quaternary combination of AZX and the mycotoxins DON, OTA, and T2. Tables S1 and S2 show the dose–effect relationship parameters (Dm, m, and r) and the mean combination index (CI) values, obtained using the resazurin and MTT methods, respectively.

The combination median–effect plot of all components and their mixtures had high regression coefficients (r), ranging from 0.92 to 0.99, as measured with the resazurin assay, and from 0.90 to 0.99 with the MTT assay. Thus, it consistently indicates adherence to the median–effect principle. The Dm (concentration required to cause a 50% effect) and m values (Hill coefficient of the dose–effect curve) were higher when measured using the resazurin method than when using the MTT method, except for the AZX + OTA + T2 and AZX + DON + OTA + T2 treatments, where no differences were found. For two-compound combinations, the rank orders based on the Dm values were AZX + T2 > AZX + DON > AZX + OTA, as shown in Tables S1 (resazurin method) and S2 (MTT method). For three compound combinations, the rank orders were AZT + DON + T2 > AZX + DON + OTA (Tables S1 and S2).

The Dm and m values obtained for individual compounds and their combinations were utilized to quantitatively measure synergistic or antagonistic effects using the CI equation. The CI quantifies the nature of the interaction between chemicals at any effect level. CI values were reported at CI10, CI25, CI50, CI75, and CI90, representing the concentration required to reach 10%, 25%, 50%, 75%, and 90% of the cells’ lethality rate, respectively. According to the IC_50_ values of the mixtures shown in Tables S1 and S2, the combination of AZX with all three mycotoxins had the highest toxic effect on HepG2 viability, with CI50 values of 0.003 and 0.006 using the resazurin and MTT assays, respectively. The lowest effect was observed for AZX + DON + T2, with CI50 values of 4.72 using the resazurin assay and 4.94 using the MTT assay. The rest of the chemical mixtures showed CI50 values between 1.67 and 2.70 according to the resazurin assay and between 1.25 and 3.79 according to the MTT assay.

**Figure 6:**
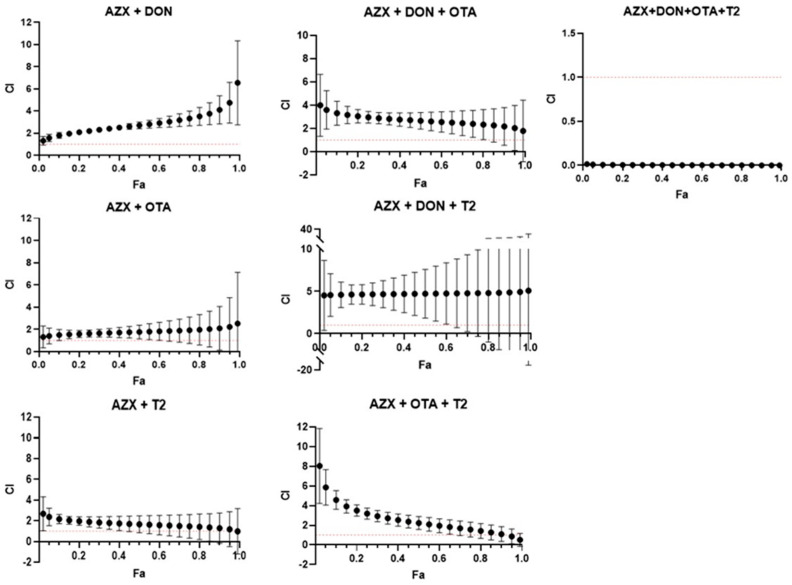
Combination index–fraction affected plots (CI-fa plot) of the tested mixtures for resazurin assay as described by the Chou and Talalay model. Each point represents the CI ± SD at a fractional affected (fa) value as determined in the cytotoxicity experiments. The dashed line (CI = 1) represents additivity, the area below the line indicates synergism (C < 1), and the area above the line indicates antagonism (C > 1). All compounds used in the combinations were in equimolar proportions. AZX: azoxystrobin; T2: toxin T2; OTA: ochratoxin A; and DON: deoxynivalenol.

**Figure 7:**
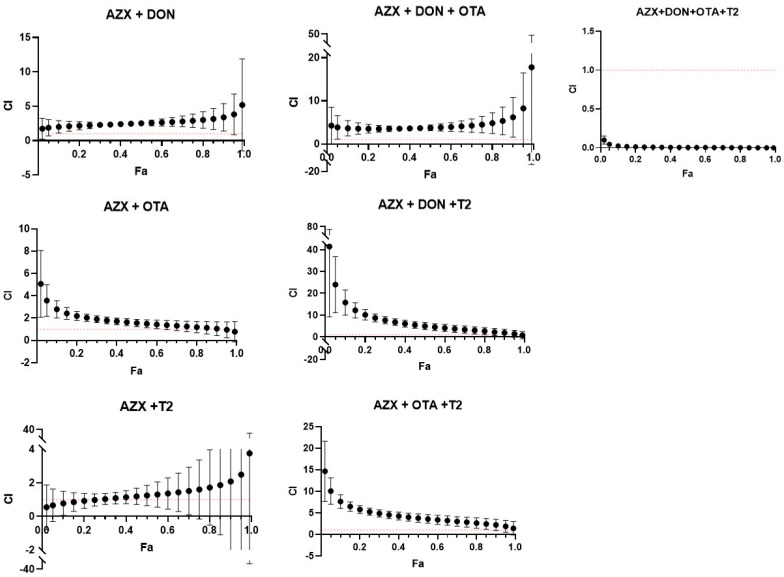
Combination index–fraction affected plots (CI-fa plot) of the tested mixtures for MTT assay as described by Chou and Talalay model. Each point represents the CI ± SD at a fractional affected (fa) value as determined in the cytotoxicity experiments. The dashed line (CI = 1) represents additivity, the area below the line indicates synergism (C < 1), and the area above the line indicates antagonism (C > 1). All compounds used in the combinations were in equimolar proportions. AZX: azoxystrobin; T2: toxin T2; OTA: ochratoxin A; DON: deoxynivalenol.

Figure 6 and Figure 7 show the combination index–fraction affected plots (CI-fa plot) for the seven mixtures analyzed of AZX and mycotoxins. CI values were plotted as a function of the fractional inhibition of cell viability (fa) using computer simulation (CalcuSyn). As assessed by the CI–isobologram equation, binary and tertiary mixtures of AZX and mycotoxins exhibited mainly antagonistic or additive effects, as determined by the resazurin and MTT cytotoxicity assays. The binary mixture AZX + DON exhibited an antagonistic effect when measured using the resazurin and MTT assays. Nevertheless, the most significant synergistic effects were observed among the most affected fractions in the mixture when employing the resazurin method. All the other binary and tertiary combinations (see Tables S1 and S2) showed antagonism at low fa values and additive effects at high effect levels. The AZX + DON + T2 mixture is an exception, demonstrating strong synergism at all the fraction levels affected. Finally, the quaternary mixture AZX + DON + OTA + T2 showed synergism at all effect levels measured under both cytotoxic methods. This phenomenon could be attributed to a common occurrence observed between combinations of substances with different mechanisms of action. The mixing of differently acting elements enhances the synergistic effect based on the dynamics of the mass action law. However, as Chou’s method does not require knowledge of the mechanisms of the compounds to establish the type of interaction that will occur, it is difficult to determine which mechanism contributes more to synergism than the others, given that the compounds in the mixture have different mechanisms [29].

## 4. Discussion

Agrochemicals and mycotoxins represent a significant dietary risk for human health. The most recent report from the Rapid Alert System for Food and Feed (RASFF) indicates that pesticide residues and mycotoxins have been consistently identified as the most prevalent hazard categories, occupying the first and third positions, respectively. Consumers can be simultaneously exposed to these compounds through various food products such as fruits, vegetables, nuts, nut products, and seeds [30], or sequentially because of mixed diets. This combined exposure to multiple chemical agents can result in interactions that alter their toxicological behavior, potentially increasing health risks [31]. However, most toxicity studies and risk assessments have focused on individual chemicals, failing to consider potential interactions between substances and leading to an underestimation of their overall impact on human health [26]. Given the potential risk of these interactions, it is crucial to evaluate the combined effects of these compounds in cellular models. This study addresses this question by examining the cytotoxicity of individual or combined exposure to AZX, a widely used active ingredient in fungicides, alongside three common mycotoxins: DON, OTA, and T2.

Two in vitro cell viability assays were employed to evaluate the acute toxicity of these compounds, as they are widely used in toxicological studies [32]. The resazurin and MTT assays were selected to utilize the complementary strengths inherent in these methodologies, thereby ensuring the successful circumvention of any limitations and a more profound understanding of cell viability [33,34]. The results of the individual toxicity tests showed that all the analyzed compounds significantly reduced cell viability in a dose- and time-dependent manner. These findings are consistent with previous results obtained using the MTT method in HepG2 cells [35,36,37]. However, differences have also been observed depending on experimental conditions, incubation time, and the cell line used [38]. In our study, the IC_50_ values revealed a clear ranking in cytotoxicity among the compounds, with T2 exhibiting the highest toxicity, followed by DON and OTA, while AZX displayed the lowest cytotoxicity. The IC_50_ of T2 was found to be in the nanomolar range, while OTA exhibited IC_50_ values approximately 150 times higher, DON around 2800 times higher, and AZX nearly 3500 times higher than T2. These findings suggest that although AZX has detrimental effects on HepG2 cells, its impact is relatively modest compared to the tested mycotoxins, particularly T2 and OTA. Moreover, the pronounced cytotoxicity observed for T2 reinforces its classification as the most toxic trichothecene. The high toxicity of this mycotoxin is linked to its chemical structure and lipophilic nature, which facilitate rapid absorption and interactions with various cells and tissues, leading to several biological effects [39]. Therefore, our findings emphasize the potential risks associated with exposure to this mycotoxin, which could have serious implications for liver health.

Notwithstanding the predominance of individual exposure in cell culture-based assessments of pesticide and mycotoxin toxicity, it is imperative to consider the impact of chemical mixtures on human toxicity, particularly in the context of dietary exposure [40]. A common method for evaluating the toxicity of chemical mixtures involves assessing the toxicity of individual components, followed by mathematical modeling to predict their combined effects [9]. Accordingly, we determined the interaction between AZX and the various mycotoxins analyzed using the CI–isobologram method developed by Chou and Talalay [28]. This is a commonly employed method in toxicology to establish the type of interaction between different components and the magnitude of this interaction [41]. Chemical interactions are typically categorized into three types: antagonistic, additive, and synergistic effects [41]. Additive effects occur when the total impact of two or more substances equals the sum of their individual effects, indicating no interaction. Synergistic effects occur when the combined impact is greater than what would be expected from simply adding the individual effects together. In contrast, antagonistic effects occur when the overall impact is less than expected [31]. The application of the CI–isobologram model helps predict additive, synergistic, or antagonistic interactions across all effect levels when combining different chemicals. In our study, combination ratios were calculated based on equipotent IC_50_ values from individual exposure experiments, ensuring an equal contribution of each compound to the overall effect. Our results revealed that the interactions between AZX and the mycotoxins analyzed varied depending on the combination and concentration, exhibiting additive, antagonistic, and synergistic patterns. The binary mixture of AZX and DON exhibited a comparable pattern in HepG2 cells across all the analyzed effect levels. The mixture demonstrated an antagonistic effect when evaluated using both the resazurin and MTT assays. Conversely, binary mixtures of AZX with OTA or T2 and tertiary combinations displayed antagonism at low fa values and additive effects at high fa values. Several key molecular factors can influence the shift from antagonistic to additive effects at different concentrations. These factors include receptor-binding dynamics, dose–response relationships, and pharmacokinetic interactions [42,43]. Additionally, feedback mechanisms and non-linear interactions can further affect how substances interact at varying concentrations, resulting in these different effects [43].

Of particular significance was the observation that the quaternary mixture of AZX, DON, OTA, and T2 exhibited a synergistic effect at all the concentration levels measured. This finding suggests that the toxicity of the assessment of cell viability of the quaternary combination is greater than that of the related individual mycotoxins. Some studies have examined the impact of the simultaneous exposure to DON, OTA, and T-2 toxins in cellular models due to their frequent co-occurrence in contaminated food and feed [38]. According to these studies, the exposure to DON and OTA or DON and T-2 show antagonistic, additive, or synergistic interactions depending on factors like cell type, toxin concentration, and exposure duration [37,44,45,46,47,48], demonstrating the complex interactions between these mycotoxins. The cytotoxic effects of the co-exposure to pesticides and mycotoxins have been studied to a lesser extent than mycotoxins mixtures. All the available data suggest that co-exposure to pesticides and mycotoxins significantly increases their toxicity, although the results vary based on the specific compounds and concentrations tested. Eze et al. (2018) studied the toxicity of DON, OTA, and other mycotoxins, such as zearalenone, on MA-10 Leydig cells, finding that their harmful effects were amplified when combined with the persistent organochlorine pesticides 1,1,1-trichloro-2,2-bis(*p*-chlorophenyl) ethane (p,p′-DDT). In contrast, combinations with its metabolite, 1,1-dichloro-2,2-bis(p-chlorophenyl) ethylene (p,p′-DDE), exhibited antagonistic effects at lower concentrations (1–8 μM) but became additive or synergistic at higher concentrations (16–64 μM) [49]. Lu et al. (2019) [50] found that the combination of chlorpyrifos (CPF) and patulin (PAT) in a fixed ratio of 1:12.5 (PAT/CPF) significantly enhanced cytotoxicity in AML12 (alpha mouse liver 12) cells. This effect exhibited a synergistic nature, as determined by the combination index calculated using the Chou–Talalay method.

Antagonistic interactions have been linked to competition among components for the same receptor, and chemicals with similar modes of action are expected to have at least additive effects [51]. On the other hand, synergistic interactions have been related to increased uptake rates through biological membranes, the formation of toxic metabolites, reduced excretion, altered distribution, and inhibited detoxification systems [52]. However, the mechanisms explaining interactions in chemical mixtures are still not fully understood and may be influenced by the mode of action of each component [53]. AZX exhibits toxicity in human cells primarily through the generation of oxidative stress, mitochondrial dysfunction, and the induction of apoptosis [54]. Exposure to DON, T-2, or OTA has also been demonstrated to induce oxidative stress, mitochondrial dysfunction, apoptosis, DNA damage, and inflammatory responses in various cell types [39,55,56]. One plausible explanation for the synergistic effect of the quaternary mixture could be the more complex composition of the mixture and the different mechanisms of action of each of the components, which may contribute to the increased cytotoxic effects. While there is a lack of research specifically examining the effects of simultaneous exposure to AZX and mycotoxins in cellular models, a previous study has analyzed the potentially toxic effects of the binary combination of AZX and OTA on various biological processes in zebrafish embryo models [25]. This study found that combined exposure to environmentally relevant concentrations of AZX and OTA caused more significant toxic effects than individual exposures, particularly in terms of oxidative stress, inducing apoptosis and altering the expression of inflammatory and thyroid hormone-related genes. Similar results were found by Wang et al. (2023), who assessed the individual and combined effects of the widely used triazole fungicide triadimefon and the naturally occurring mycotoxin citrinin on different biological processes in zebrafish embryos. As for the AZX and OTA combination, co-exposure to triadimefon and citrinin induced interactive effects on biological processes and biomarkers related to cell death, oxidative stress, inflammation, apoptosis, and the regulation of thyroid hormones [57]. Similarly, Lu et al. (2019) found that the combination of chlorpyrifos (CPF) and patulin (PAT) resulted in more significant inactivation of the enzyme catalase and an increase in the production of ROS [50]. Thus, the available data suggest that the potential mode of action responsible for the cytotoxic effects observed in the synergistic interaction between simultaneous exposure to AZX, DON, T-2, and OTA may result from common metabolic processes, such as oxidative stress and energy metabolism. Moreover, the transition from antagonistic to additive effects observed in binary and tertiary mixtures could also be related to the underlying mechanism of action of oxidative stress induction. At low concentrations, the organism’s antioxidant defense mechanisms can effectively counteract oxidative stress by synthesizing both antioxidant and biotransformation enzymes. These enzymes work together to prevent ROS accumulation in adverse conditions, leading to antagonistic effects. However, these defense mechanisms may become overwhelmed at higher concentrations of chemicals or mixtures, and the antioxidant system may be insufficient to handle environmental stress. This can result in additive or synergistic effects [58].

Our results reinforce the necessity of examining specific pesticides and mycotoxin combinations to understand better the toxicological risks posed by simultaneous exposure to these compounds. However, the results are challenging to interpret because the isobologram method does not provide insight into the action mechanism of each compound in the mixture. Consequently, further research is imperative to elucidate the specific metabolic pathways involved in these interactions and to assess their potential implications for human and animal health. Current research on chemical interactions faces several key gaps, including a limited understanding of the underlying molecular mechanisms, reliance on simplified in vitro models, challenges in extrapolating in vitro data to in vivo contexts, and a lack of chronic exposure studies [59]. To address these issues, it is necessary to integrate pathway-based toxicity evaluations; alternative cellular models, such as primary hepatocytes and 3D organoid systems; computational models; multi-omics approaches; and conducting long-term in vivo studies to better understand the toxicological impact and identify biomarkers for assessing health risks [60,61]. A lack of understanding regarding the interactions between pesticides and mycotoxins can result in inaccurate risk assessments concerning the safety of food products. In real-world situations, consumers may be exposed to both pesticides and mycotoxins through contaminated food, with exposure levels fluctuating based on agricultural practices, food processing methods, and dietary habits [62,63]. By examining how these substances interact at the molecular level, we can improve risk assessments and set more precise safety thresholds for combined exposures. This could lead to stricter regulations on pesticide residues and mycotoxin levels in food. This knowledge can also enhance risk management strategies, based on scientific evaluations, for foods that significantly contribute to dietary exposure. By utilizing this information, both regulators and the industry can work together to maintain contaminant levels as low as possible.

## 5. Conclusions

Agrochemicals and naturally occurring toxins in plants can create complex exposure situations. The results of this study revealed that simultaneous exposure to AZX and DON, OTA, or T-2 results in interactions between them, modifying their toxicological behavior. These modifications depend on the specific components and their concentrations within the mixtures.

The synergistic effects observed for the quaternary combination of AZX with the mycotoxins DON, OTA, and T2 indicate a potential risk related to dietary co-exposure to these chemicals in complex mixtures. These results highlight that the traditional method of evaluating the toxicity of individual components may lead to an underestimation. Further research is recommended to examine the combined toxicities of pesticides and other xenobiotics, including mycotoxins. This would provide a more comprehensive understanding of the potential health risks associated with exposure to these mixtures.

## Figures and Tables

**Table 1:** Concentrations used and combination ratios assayed to determine the toxicological interactions of azoxystrobin (AZX) and their binary, ternary, and quaternary combinations with deoxynivalenol (DON), ochratoxin A (OTA), and T-2 toxin (T2).

	Concentration (µM)				
	IC_50/8_	IC_50/4_	IC_50/2_	IC_50_	Ratio
AZX	25.76	51.53	103.05	206.10	1
DON	1.17	2.35	4.70	9.39	0.05
OTA	24.41	48.83	97.65	195.30	0.95
T2	0.007	0.015	0.030	0.059	0.0003

IC_50_: mean inhibition concentration; AZX: azoxystrobin; DON: deoxynivalenol; OTA: ochratoxin A; and T2: T-2 toxin.

## Data Availability

The original contributions presented in the study are included in the article/supplementary material, further inquiries can be directed to the corresponding author.

## Supplementary Materials

The following supporting information can be downloaded at https://www.mdpi.com/article/10.3390/foods14071226/s1, Table S1: Dose–effect relationship parameters and mean combination index (CI) values of the binary, tertiary, and quaternary mixture of AZX, DON, OTA, and T2 on HepG2 cells using the resazurin method; Table S2: Dose–effect relationship parameters and mean combination index (CI) values of the binary, tertiary, and quaternary mixture of AZX, DON, OTA, and T2 on HepG2 cells using the MTT method.

## Abbreviations

The following abbreviations are used in this manuscript:

AZX	azoxystrobin
CI	combination index
Dm	median–effect concentration
DMEM	Dulbecco’s Modified Eagle Medium
DMSO	dimethyl sulfoxide
DON	deoxynivalenol
EFSA	European Food Safety Authority
IC_50_	mean inhibition concentration
MRL	maximum residue level
MTT	thiazolyl blue tetrazolium bromide
NBCS	Newborn Calf Serum
OTA	ochratoxin A
PBS	Phosphate-Buffered Saline
RASFF	Rapid Alert System for Food and Feed
SEM	standard error of the mean
T2	T-2 toxin
