# Supplementary material for: Combined Cytotoxic Effects of the Fungicide Azoxystrobin and Common Food-Contaminating Mycotoxins
Source: Foods. 2025 Mar 31;14(7):1226. doi: 10.3390/foods14071226 (PMC11988556; doi:10.3390/foods14071226)
Supplement: Supplementary file 1 [file foods-14-01226-s001.zip › foods-3528521-supplementary.pdf]

foods

Combined cytotoxic effects of the fungicide Azoxystrobin and common food-contaminating mycotoxins

Cristina Fuentes[1,2*], Veronica Zingales[2,3], José Manuel Barat[1], María-José Ruiz[2,3]

[1]University Institute of Food Engineering–FoodUPV, Universitat Politècnica de València. Camino de Vera s/n, 46022, Valencia, Spain: *crifuelp@upvnet.upv.es

[2]Research group in Alternative methods for determining toxics effects and risk assessment of contaminants and mixtures (RiskTox).

[3]Laboratory of Food Chemistry and Toxicology, Faculty of Pharmacy, Universitat de València, Av. Vicent Andrés Estellés s/n, 46100 Burjassot, València, Spain

Table S1. Dose–effect relationship parameters and mean combination index (CI) values of the binary, tertiary, and quaternary mixture of AZX, DON, OTA, and T2 on HepG2 cells using the resazurin method.

Treatment	*Dm* (μM)	m	r	CI values				
				CI_{10}	CI_{25}	CI_{50}	CI_{75}	CI_{90}
**AZX**	637.98	0.99	0.9719	–	–	–	–	–
**DON**	5.33	0.90	0.9933	–	–	–	–	–
**OTA**	87.57	1.03	0.9613	–	–	–	–	–
**T2**	0.10	0.91	0.9198	–	–	–	–	–
**AZX+DON**	309.89	0.78	0.9988	1.79 (0.27)	2.20 (0.18)	2.70 (0.30)	3.33 (0.68)	4.11 (1.27)
				Ant	Ant	Ant	Ant	Ant
**AZX+OTA**	265.73	0.95	0.9663	1.50 (0.49)	1.63 (0.31)	1.77 (0.62)	1.93 (1.19)	2.10 (1.97)
				Ant	Ant	Ant	Add	Add
**AZX+T2**	465.18	1.06	0.9823	2.17 (0.46)	1.92 (0.48)	1.67 (0.81)	1.47 (1.14)	1.30 (1.46)
				Ant	Ant	Add	Add	Add
**AZX+DON+OTA**	192.46	1.05	0.9613	3.32 (1.04)	2.97 (0.50)	2.66 (0.71)	2.40 (1.16)	2.17 (1.63)
				Ant	Ant	Ant	Ant	Add
**AZX+DON+T2**	439.19	0.91	0.9219	4.60 (1.51)	4.66 (1.34)	4.72 (2.89)	4.79 (5.10)	4.87 (8.10)
				Ant	Ant	Ant	Add	Add
**AZX+OTA+T2**	256.34	1.47	0.9811	4.57 (0.95)	3.18 (0.56)	2.22 (0.67)	1.56 (0.75)	1.10 (0.76)
				Ant	Ant	Ant	Add	Add
**AZX+DON+OTA+T2**	0.20	1.55	0.9983	0.008 (0.000)	0.005 (0.000)	0.003 (0.000)	0.002 (0.001)	0.001 (0.001)
				Syn	Syn	Syn	Syn	Syn

CI < 1, CI = 1, and CI > 1 indicate synergism (Syn), additive effect (Add), and antagonism (Ant), respectively.

Table S2. Dose–effect relationship parameters and mean combination index (CI) values of the binary, tertiary, and quaternary mixture of AZX, DON, OTA, and T2 on HepG2 cells using the MTT method.

Treatment	*Dm* (µM)	m	r	CI values				
				CI_{10}	CI_{25}	CI_{50}	CI_{75}	CI_{90}
**AZX**	283.88	0.81	0.9756	–	–	–	–	–
**DON**	2.74	0.69	0.9916	–	–	–	–	–
**OTA**	57.14	1.26	0.9603	–	–	–	–	–
**T2**	0.06	0.94	0.9258	–	–	–	–	–
**AZX+DON**	144.60	0.63	0.9871	2.01 (0.90) Ant	2.23 (0.48) Ant	2.52 (0.34) Ant	2.89 (0.97) Ant	3.39 (1.99) Ant
**AZX+OTA**	137.94	1.40	0.9756	2.79 (0.77) Ant	2.03 (0.34) Ant	1.56 (0.34) Ant	1.26 (0.48) Add	1.06 (0.62) Add
**AZX+T2**	178.95	0.73	0.8955	0.78 (0.71) Add	0.98 (0.37) Add	1.25 (0.60) Add	1.61 (1.76) Add	2.08 (4.23) Add
**AZX+DON+OTA**	148.07	0.73	0.9739	3.64 (1.79) Ant	3.52 (0.80) Ant	3.73 (0.68) Ant	4.49 (1.92) Ant	6.19 (4.61) Ant
**AZX+DON+T2**	236.43	1.11	0.9550	15.75 (5.81) Ant	8.67 (1.85) Ant	4.94 (1.56) Ant	2.93 (1.65) Ant	1.82 (1.63) Add
**AZX+OTA+T2**	256.34	1.47	0.9811	7.64 (1.57) Ant	5.27 (0.84) Ant	3.79 (0.97) Ant	2.84 (1.16) Ant	2.21 (1.31) Add
**AZX+DON+OTA+T2**	0.20	1.55	0.9983	0.026 (0.010) Syn	0.012 (0.001) Syn	0.006 (0.001) Syn	0.003 (0.001) Syn	0.002 (0.001) Syn

CI < 1, CI = 1, and CI > 1 indicate synergism (Syn), additive effect (Add), and antagonism (Ant), respectively.
